# Optimism and the Psychological Recovery Process Among Informal Caregivers of Inpatients Suffering From Depressive Disorder: A Descriptive Exploratory Study

**DOI:** 10.3389/fpsyt.2019.00972

**Published:** 2020-01-16

**Authors:** Claire Coloni-Terrapon, Jérôme Favrod, Aurélie Clément-Perritaz, Isabelle Gothuey, Shyhrete Rexhaj

**Affiliations:** ^1^La Source, School of Nursing Sciences, HES-SO University of Applied Sciences and Arts Western Switzerland, Lausanne, Switzerland; ^2^Fribourg Network for Mental Health (FNPG), Marsens, Switzerland; ^3^School of Nursing Sciences, HES-SO University of Applied Sciences and Arts Western Switzerland, Fribourg, Switzerland

**Keywords:** informal caregivers, depressive disorders, psychological adaptation, nursing, recovery

## Abstract

**Background:** Informal caregivers of people suffering from depressive disorders go through a psychological recovery process. This process is dynamic, deep, catalyzed by hope and optimism and characterized by stages from which specific needs ensue. This study aimed to describe the stages of the psychological recovery process and the level of optimism among informal caregivers of psychiatric inpatients suffering from depressive disorders in order to provide adapted nursing support and psychoeducation and facilitate a patient's own recovery.

**Methods:** A descriptive exploratory study was conducted using a convenience sample of 29 informal caregivers. Participants filled out a sociodemographic questionnaire, a specially adapted Stages of Recovery Instrument (STORI) and the Life Orientation Test–Revised (LOT–R).

**Results:** A mean optimism score of 16.41 showed that informal caregivers are close to the level of the general European population. The sample included all the stages of the recovery process, with 34.5% of participants being in the growth stage. Informal caregivers' stages in the recovery process were negatively associated with the patient's length of illness (Rho = -.683, p = .000) and positively associated with the caregivers' level of optimism (Rho = .564, p = .001).

**Conclusion:** During the inpatient treatment of a close relative suffering from a depressive disorder, informal caregivers go through an individual psychological recovery process involving several stages. In addition to caring for inpatients, nurses are encouraged to meet and support caregivers as soon as possible in their individual recovery process. Furthermore, the development of a suitably adapted clinical tool would facilitate the assessment of the informal caregiver's stage in the recovery process within care units. A multidisciplinary approach is needed in this domain.

## Introduction

The proportion of the global population suffering from depression in 2015 was estimated to be 4.4% (1). Depression is the leading cause of disability worldwide and is a major contributor to the overall global burden of disease (2). The annual prevalence of major depression, established by epidemiological research, is 7% (3). In Swiss psychiatric institutions, depressive disorders are the most frequently treated diagnosis (4). Indeed, they represent approximately 26% of psychiatric inpatient diagnoses and remain the most common reason for hospitalization among women (3). The considerable consequences of depressive disorders affect not only sufferers but also their close relatives. Shorter hospital lengths of stay, as well as the current trend in mental health care systems of keeping people suffering from psychiatric disorders in the community and making families become part of care provision, can considerably increase the burden and responsibilities placed on informal caregivers (5).

Informal caregivers can experience the psychiatric hospitalization of the depressive patients as either a relief or as a traumatic event which gives rise to shame, guilt, and worry (6, 7). Caregivers have described lower activity levels and experiencing feelings of loss and worry about the future, social isolation and a lack of support from family, friends, and health care professionals, particularly during the acute phase of a patient's illness (8, 9). Those same caregivers sometimes have to struggle to become involved in the patient’s care and be listened to by health care professionals (10). According to Van der Sanden et al., 74% of the informal caregivers of persons suffering from psychiatric illnesses, mainly depressive disorders, report having experienced stigmatization, which affected their own well-being and social and professional lives (11). In another study, more than half the caregivers described a feeling of being held responsible for the illness, being rebuked or being excluded from the patient's care by health care professionals (12). It’s a noteworthy paradox that sometimes informal caregivers do not feel relieved when caring for someone with a mental health problem. The literature indicates that the hospitalization period seems to be a crucial moment at which professionals could provide support and psychoeducation to reduce the informal caregivers' level of distress and burden (13–17). However, most of the evaluations and psychoeducation of informal caregivers in a context of major depression is given within a specified program rather than care units (18).

Some studies have revealed a psychosocial process experienced by the close relatives of patients with a depressive or other mental disorder (19–27). These different results show that this process begins by a first stage involving a lack of knowledge about the patient's disease. This stage generates feelings of helplessness and worry as depressive symptoms appear or the patient risks becoming suicidal (21–24). The next stage involves the recognition of the serious condition, engendering guilt, sadness, despair, a feeling of loss and a search for help (19, 21–25). The illness's impact on loved ones is then highlighted: it touches every facet of the caregiver's life until it absorbs their identity or leads them to repress their own needs (19, 20, 22–24, 27). These elements bring informal caregivers into the next stage, which is the adaptation stage. Different results have revealed a reorganization of family roles: the use of adaptive strategies within a process of trial and error to adapt to the needs of the patient suffering from a depressive disorder (19–24, 27). The different challenges confronting informal caregivers illustrate the following stage: they seek the recognition of health care professionals and wish to be listened to in order to overcome the stigma of mental illness (19, 20, 23–25). The final stage of the process involves the caregiver's will to move on. This means a search for balance and the power to act, a transformation to a new self, a hope of a better future, a re-evaluation of one's own values and a solidarity with families living the same situation (19, 20, 22–25, 27). It appears that informal caregivers experience a constant burden throughout the process, even though their personal sense of control tends to increase (26).

Retta Andresen and her colleagues defined the psychological recovery process as “the establishment of a fulfilling, meaningful life and a positive sense of identity founded on hopefulness and self-determination” (24 p. 588). According to the same authors, the psychological recovery process comprises four essential attributes: the presence and maintenance of hope, the establishment of a positive identity, the meaning one gives to one's life, and taking responsibility for one's own recovery (28, 29). In addition, they identified five stages in the recovery process, beginning with a denial of the illness, confusion about one's identity, withdrawal from society, or despair at ever rediscovering any meaning in one's life, a positive perception of self and optimism about the future, despite the persistence of symptoms. The stages are moratorium, awareness, preparation, rebuilding, and growth (28, 29).

Although optimism and hope are not redundant constructs, they are positively related to each other and positively related to psychological and physical well-being (30). Hope focuses more directly on the personal attainment of specific goals, whereas optimism focuses more broadly on the expected quality of future outcomes in general (31).

It seems essential to adopt a relational vision of recovery (32). When a mental illness appears, informal caregivers experience a traumatic event that affects every aspect of their lives. Just like patient, they undergo a recovery process tending towards a life that includes hope and new goals for the future (33, 34). The literature indicates that family play a key role in supporting the patient and can both facilitate or impede a patient's recovery process (35, 36). Therefore, supporting informal caregivers requires a recognition of their recovery stage to inspire hope and offer quality, individualized care, psychoeducation and support throughout the patient's psychiatric illness (17, 34).

Unfortunately, the scientific literature contains very little information on informal caregivers' process of psychological recovery. No quantitative studies have been done until then, especially in the context of a psychiatric disorders (33, 34). If the association between optimism and recovery is well documented for people suffering from a mental illness, it is not for informal caregivers (29, 37, 38).

Thus, the present exploratory study proposes to 1) describe the stages of the psychological recovery process in which the informal caregivers of depressive inpatients find themselves, 2) measure their level of optimism and 3) explore the association between stages of recovery, optimism, and sociodemographic factors.

## Materials and Methods

### Design and Recruitment

This descriptive exploratory study was conducted using 29 informal caregivers of patients suffering from a depressive disorder and hospitalized in a psychiatric inpatient or day-hospital setting. The term *informal caregivers* can imply family members, friends or any other significant individuals who consider themselves concerned by or involved with the patient and who gives that patient support or company (39). Data collection took place in two adult general psychiatry units and two day-hospital units in an institution in French-speaking Switzerland over a period of six months from October 2016 to March 2017.

The informal caregivers were recruited via a non-probabilistic convenience sampling method. Their recruitment was effected through the patients using the following inclusion criteria: 1) patients aged between 18 and 65 years old 2) whose main diagnosis or reason for admission was suffering from a depressive disorder (DSM-V: F.32-33), 3) who were hospitalized in an inpatient or day-hospital setting, and 4) who were capable of discernment. The exclusion criterion was the refusal to include an informal caregiver.

Patients meeting the criteria were identified on admission and given oral and written information about the project by the care team. When patients gave their agreement, they were asked to identify a close family member or friend whom they believed helped them the most. The inclusion criteria for the informal caregivers were as follows: 1) at least 18 years old, 2) capable of reading and speaking French, and 3) being an informal caregiver without minimum duration of support. There were no exclusion criteria.

Eligible informal caregivers were also given oral and written information by the care team. If they, in turn, agreed to participate, they were contacted by telephone by the principal investigator in order to arrange a meeting during the patient's hospitalization. Interviews took place in a hospital meeting room or at caregivers' home or workplace.

### Instruments

Participants filled out a) a self-administered sociodemographic questionnaire and two measure instruments: b) the Life Orientation Test–Revised (LOT–R) and c) the Stages of Recovery Instrument (STORI), adapted for informal caregivers.

#### Sociodemographic Questionnaire

A self-administered questionnaire was used to collect informal caregivers' sociodemographic data: age, sex, level of education, civil status, number of persons living in the home, employment status and percentage of full-time employment, relationship with the patient, frequency of contact, and patient's length of illness.

#### The Life Orientation Test—Revised

Informal caregivers' level of optimism were measured using the LOT–R validated in English by Scheier et al. (40, 41). Optimism refers to an individual's positive expectations when faced with a given situation (state optimism) or with life in general (trait optimism) (42). LOT–R comprises six personal evaluation statements and four decoy statements. The personal evaluation statements deal specifically with people's general expectations with regards to positive consequences (three statements) or negative consequences (three statements). LOT–R thus enables a self-evaluation of one's optimism using a five-point Likert scale running from 0 (totally disagree) to 4 (totally agree). The total evaluation of optimism score is obtained by adding the three positive statement scores to the three inversed negative statement scores. The decoy statements are not counted towards the final score. LOT–R results can thus range from 0 to 24 (41). A 2008 French-language version of LOT–R was validated using 208 Francophone university students. This demonstrated that the French-language version possessed satisfactory psychometric properties comparable with those of the English-language version (Cronbach's alpha, 0.76; test-retest reliability, 0.74) (39, 43).

#### The Stages of Recovery Instrument (Adapted for Informal Caregivers)

The informal caregivers' stage of psychological recovery process was revealed by the “Stages of Recovery Instrument”, adapted for informal caregivers (STORI for informal caregivers). Retta Andresen developed the STORI in 2006, and Golay and Favrod developed the French version in 2008 (29, 44). The scale was adapted for informal caregivers in 2016 using the consensus decisions of a panel of 12 informal caregivers helping persons suffering from mental illnesses. The scale comprises 50 items presented in ten groups. Each group represents one of the four essential components of recovery: hope, identity, meaning, and responsibility. There are two or three groups to each component. The five individual items comprising each group represent sub-scales and correspond to the five stages of recovery: moratorium, awareness, preparation, rebuilding, and growth. Participants evaluate each item using a six-point Likert scale running from 0 (not at all true now) to 5 (completely true now). The total score for the first items in each group gives the score for stage 1, the total score for the second items in each group gives the score for stage 2, and so on. The recovery stage with the highest score is retained as the informal caregiver's current stage. If two stages share the highest score, the more advanced stage in the recovery process is retained (29). The STORI scale, aimed at patients, shows good reliability for each of the five sub-scales (Cronbach's alpha from 0.88 to 0.94) (29).

### Data Analysis

Data analysis was carried out using STATA statistical software, version 14. Descriptive statistical analyses were used to describe informal caregivers' sociodemographic characteristics, level of optimism, and stage of psychological recovery process. Spearman's rank correlation coefficient, the Mann–Whitney U test, and Kruskal-Wallis test were used to measure correlations and differences among the stage of recovery, the level of optimism, and a range of other informal caregivers' variables. The results with a p-value below 0.05 were considered statistically significant.

### Ethical Considerations

The research protocol received full authorization from the Human Research Ethics Committee of the Vaud Canton. All participants were informed about the study and their rights and signed a written informed consent form.

## Results

### Sample Characteristics

On 82 eligible patients, 25 refused to participate in this study (a 69.5% response rate). Of the 57 patients who agreed to participate, 13 did not mention any informal caregiver and 4 refused the informal caregiver's participation. Of 40 eligible informal caregivers, 11 refused to participate (a 72.5% response rate). Refusal reasons are unknown.

**Table 1** presents the study participants' sociodemographic characteristics. The final sample comprised 29 informal caregivers: 20 women (68.97%) and 9 men (31.03%). Participants' mean (M) age was 42.62 years old with a standard deviation (SD) of 15.11 (women: M = 40.8, SD = 15.8 men: M = 46.6, SD = 14.27). Sixteen (55.17%) informal caregivers cohabited with the patient and 19 (65.52%) had professional activities, ranging from 25% to 100% full-time employment. More than half (n = 10) were working at least full-time. The majority of informal caregivers were spouses or children of the patient (55.18%), had daily contact with the patient (72.41%) and estimated their income as medium (62.1%). Of the 29 patients who facilitated the recruitment of the informal caregivers, 72.41% (n = 21) were inpatients and 27.59% (n = 8) were being treated in a day-hospital context. The mean of the patient's length of illness was 10.30 years (SD 13,94), ranging from 1 to 38 years. Most of the patients had a length of illness from 1 to 5 years (see **Table 2**).

**Table 1:** Sociodemographic characteristics of informal caregivers (n = 29).

Variables	N	%	Mean (SD)	Min–Max
**Informal caregiver's sex**				
Women	20	68.97		
Men	9	31.03		
**Informal caregiver's age**	29		42.62 (15.11)	18-71
Women	20		40.8 (15.48)	18-66
Men	9		46.6 (14.27)	28-71
[18-30]	8	27.6		
[30-45]	8	27.6		
[45-65]	10	34.5		
[65-71]	3	10.3		
**Informal caregiver's cohabitation with patient**				
Yes	16	55.17		
No	13	44.83		
**Informal caregiver's professional activity**				
Yes	19	65.52		
No	10	34.48		
**Percentage of full-time employment in professional activity**	19		81.32 (27.28)	25-120
**Informal caregiver's relationship to patient**				
Father/mother	7	24.14		
Son/daughter	8	27.59		
Brother/sister	4	13.79		
Spouse/partner	8	27.59		
Friend m/f	2	6.9		
**Informal caregiver's level of education**				
Completed obligatory schooling	7	24.14		
Apprenticeship	9	31.03		
High-school or secondary school diploma	3	10.34		
Professional school	7	24.14		
University	3	10.34		
**Informal caregiver's civil status**				
Single	11	37.93		
Married/living with partner	13	44.83		
Widower/widow	1	3.45		
Separated/divorced	4	13.79		
**Informal caregiver's estimated income**				
Insufficient	3	10.3		
Low	5	17.2		
Medium	18	62.1		
High	3	10.3		
**Frequency of contact between informal caregiver and patient**				
At least 1 h/month	1	3.45		
1 to 3 times/week	5	17.24		
4 to 6 times/week	2	6.9		
Every day	21	72.41		

**Table 2:** Sociodemographic characteristics of patients (n = 29).

Variables	N	%	Mean (SD)	Min–Max
**Patient's sex**				
Women	19	65.52		
Men	10	34.48		
**Patient's age**	29		43.52 (12.22)	19-61
Women	19		44.58 (11.75)	19-58
Men	10		41.5 (13.48)	21-61
**Place of care**				
Hospital ward	21	72.41		
Day-hospital	8	27.59		
**Length of illness**	29		10.30 (13.94)	1-38
[1-5]	18	62.1		
[5-15]	4	13.8		
[15-30]	2	6.9		
[30-38]	5	17.2		

### Level of Optimism

Results for informal caregivers' level of optimism ranged from 6 to 24 (M = 16.41; SD = 4.63), with a median of 17.

### Stages of Psychological Recovery Process

Results showed that only one informal caregiver was at the moratorium stage; 13.8% (n = 4) were at the awareness stage, 20.7% (n = 6) at the preparation stage and 27.6% (n = 8) at the rebuilding stage. The final recovery stage of growth was the most well represented, with 34.5% (n = 10) of the informal caregivers.

A statistically significant negative association (Rho = -.683, p = .000) was found between the caregivers' stage of psychological recovery process and patients' length of illness. Longer patient illness length was associated with a less advanced caregivers' stage of psychological recovery process. No other association between informal caregivers' stage and sociodemographic data was found (see **Table 3**).

**Table 3:** All statistical tests for associations between informal caregivers' stages of psychological recovery and their sociodemographic data (n = 29).

Variables	Stages of psychological recovery	
**Age**	Rho = .062	*P* = .747
**Sex**	Z = 0.539	*P* = .590
**Cohabitation**	Z = -0.114	*P* = .909
**Professional activity**	Z = -1.644	*P* = .100
**Percentage of activity**	Z = 0.147	*P* = .883
**Relationship**	X² = 6.731	*P* = .151
**Level of education**	Rho = .203	*P* = .292
**Civil status**	X² = 2.500	*P* = . 475
**Frequency of contact**	Rho = .045	*P* = .816
**Context for hospitalization**	Z = 0.811	*P* = .417
**Length of illness**	Rho = -.683	P = .000

Rho = Spearman, Z = Mann-Whitney, X² = Kruskal–Wallis.

Lastly, we found a statistically significant positive association (Rho = .564, p = .001) between the stage of psychological recovery process and the informal caregivers' level of optimism. A higher level of optimism was associated with a more advanced stage in the psychological recovery process (see **Table 4**).

**Table 4:** Associations between informal caregivers' stages of psychological recovery and their level of optimism (n = 29).

LOT-R	stage 1 (n = 1)	stage 2 (N = 4)	stage 3 (N = 6)	stage 4 (N = 8)	stage 5 (N = 10)
**MEDIAN (SD)**	14 (-)	14 (5.802)	14 (5.279)	17 (3.576)	19 (3.062)
**IQR**	0	8	7	4	5

## Discussion

To the best of our knowledge, the present study is the first to examine the informal caregivers' level of optimism and stage of psychological recovery process when their close relatives, suffering from a depressive disorder, are admitted to a psychiatric inpatient or day-hospital unit.

The present results showed that informal caregivers had a level of optimism mean score of 16.41. Interpretation of these results does not enable categorization of caregivers as optimistic or pessimistic, but the mean of 16.41 suggests that the participants are relatively close to the level of optimism of the general European population (45, 46). A study conducted in 2017 with a German general community sample showed a mean score of 16.2 (45). According to Schou-Bredal et al., who administered the LOT–R to a representative population-based sample in Norway, the mean score was 17.2 (46). The study found that a higher level of optimism was associated with a higher level of perceived health and quality of life (46). It is interesting to note that in the present results, the caregivers' mean optimism score is below the European average in the three first stages of the process. The two last stages (rebuilding and growth) show results above the average. The literature indicates that the difference between pessimistic and optimistic persons can be seen in how they adapt to life events (40, 41). According to Scheier & Carver, optimists seem more likely to use adaptive strategies centered on the problem itself or turn to emotional strategies such as acceptance, humour or positive reframing. The different ways optimists and pessimists approach the world appear to have a substantial impact on their respective lives (40, 41). According to Priestley & McPherson, adapting to depression for informal caregivers encompass a sense of integrating the depression into the relationship and family life and means accepting the realities and limits of their adapted life (19).

The results showed that all the stages of the recovery process were represented, with most of the informal caregivers found in the next-to-last (rebuilding) and last (growth) stage. Informal caregivers go through this psychological recovery process in several stages, and they could be in any one of these stages when their patient is hospitalized for a depressive disorder. Andresen et al.'s theoretical framework for recovery shows that it is once the informal caregiver reaches the rebuilding stage that the hard work of recovery truly begins. In this stage, informal caregivers tend to forge themselves positive new identities and move forward to the completion of personal goals and toward new values (28). This stage encompasses taking responsibility for and control over one's own life, despite potential relapse, often experienced in the depressive disorder (17, 19, 28, 29, 47). In order for informal caregivers to move forward, they need to accept that life cannot return to how it was prior to the patient's depression (19). The fifth and final stage can be considered the result of a fully completed process of psychological recovery (28). This means that 34.5% of the informal caregivers included in the study could be considered as having recovered. In this final stage, informal caregivers have confidence in their abilities—they maintain a positive outlook, turned towards the future. Despite the possible chronic or recurrent character of depression and the suicidal risk by their ill relative, they can preserve their own health (23). They can perceive that they had become more resilient (48). They feel transformed by the life experience of dealing with their patient's illness (17, 28, 29, 47). They may wish to use their experience to support their caregiving peers (26, 29). Depression is now integrated into the family life (19). However, these results may be explained by the fact that the further advanced informal caregivers are in their process of recovery, the more likely they are to share their experience and be motivated to participate in the study. No comparable studies were available to validate these results; however, two studies using the STORI with persons suffering from severe mental illnesses evidenced similar results: all the stages of psychological recovery were represented, and the majority of participants were to be found in the final two stages of the process (29, 49).

The present study also demonstrated that the patient's length of illness was associated with the process of psychological recovery undergone by informal caregivers. A patient's shorter length of depression was associated with a more advanced caregivers' stage of psychological recovery process. This relationship may seem surprising at first. Indeed, according to Spaniol & Nelson, recovery is a painful and deeply emotional process of self-discovery, self-renewal, and transformation (34). It involves informal caregivers facing reality and readjusting their attitudes, feelings, perceptions, and beliefs. It also requires building new connections to oneself, to others, to life (34). This ongoing process seems likely to be considered in the long term. Furthermore, the literature on recovery undergone by people suffering from mental illness seems to show that duration of illness, age at onset of illness or duration since diagnosis have no significant correlation with the STORI (29, 50). However, this result maybe highlights the specificity of living with a person suffering from depression. In a study about emerging adults living with a parent suffering from depression, the results showed that the length or chronicity of the parent's depressive symptoms was associated with a lesser psychosocial well-being (51). In another study about the experience of living with a partner with chronic depression, some participants described their difficulties to maintain the same level of empathy toward their partner during long periods of depression or during subsequent episodes (27). Moreover, according to Nosek, the informal caregivers' psychosocial process is cyclical by nature, because informal caregivers often experience depression as a recurrent and unstable condition (22). Likewise, Priestley & McPherson identify that relapses can take the informal caregivers back to a previous stage of the process (19). However, they can pass through the stages more rapidly, being more experienced at dealing with depression (19).

Lastly, the positive association between the stages of psychological recovery and the level of optimism found in this study is not surprising. Indeed, hope and optimism are omnipresent in the literature on recovery (10, 19, 23, 25, 29, 33, 34, 52–56). According to Andresen et al.'s theoretical framework on psychological recovery, hope is one of the essential and fundamental components of this process and grows throughout the process (28, 29). Hope is a catalyst which can come from within the person or be instilled by a significant relation or a peer (28). Maintaining hope seems to be a complex issue for informal caregivers, however. First and foremost, it signifies maintaining hope for the patient (25). This implies managing the uncertainties surrounding the patient's prognosis—a task which is particularly difficult in the early stages of the illness when hope is often vacillating and fragile and the feeling of loss is strong (56). According to Priestley & McPherson, even if caregivers can adapt to depression, they look to the future with hope but also apprehension due to the unpredictability of depression (19). To better support informal caregivers in their process of psychological recovery, individualized brief programs are recommended (39).

### Limitations of the Study

This study has several limitations. First, the inclusion criteria created a complex selection process requiring the patient's agreement before attempting to recruit the informal caregiver. This process explains the small sample and has further strengthened the presence of the most motivated and close dyads of patients and relatives. The process and the convenience sampling may constitute two important biases that may prevent the generalization of our results.

A second limitation concerns interviews and questionnaires with informal caregivers that were carried out between the 8th and 25th days of the patient's hospitalization. This wide range is due to informal caregivers' busy daily lives and limited availability. However, this gap may have affected our results with heterogeneity of time recovery.

Third, the STORI for informal caregivers has yet to be validated empirically. This methodological weakness was reduced by the use of the LOT–R, a complemented scale with good psychometric properties.

Lastly, confounding variables related to patients such as severity of depression, comorbidities as well as other personal characteristics may have influenced the results. Some confounding variables related do informal caregivers' such as burden, quality of life or personal level of health may have also affected the results.

## Conclusions

This exploratory study proposes a first highlight of the psychological recovery process undergone by caregivers in a context of depressive disorder and its influencing factors. The results showed that informal caregivers are situated at different stages of the process. When their close relative suffering from a depressive disorder is hospitalized, they may be at the beginning or at the end of that process. The main factors associated with the recovery process were the patient's length of illness and the caregivers' level of optimism.

### Implications for Practice

A tailored early intervention and support for informal caregivers should be encouraged to facilitate their process of psychological recovery, in coordination with others health care professionals, during hospitalizations (57). Furthermore, it should consider the patient's length of illness, the potential chronicity of the depressive disorder and the caregiver's level of optimism. Indeed, such an intervention could significantly improve the informal caregiver's psychological health and thus facilitate the patient's own recovery. In addition, we recommend the development of an adapted clinical tool for the evaluation of informal caregivers' stages of psychological recovery process within care units. Such an instrument would help health care professionals offer care specifically adapted to the needs of informal caregivers.

### Implications for Research

This topic deserves further empirical investigations. In order to limit potential biases and increase the sample size, studies should use a simpler selection process, focusing on caregiver's health, independently of the patient's participation in the research. However, study interested in both patients' and their caregivers' recovery process are needed do better describe the interactions for each other. It would also be necessary to integrate other informal caregivers' variables, measuring psychological characteristics, quality of live and burden in order to better control confounding variables. Additional semi-directive interviews could also help deepen this topic in order to provide more in-depth understanding of the informal caregivers' recovery processes experiences. Finally, we recommend the empirical validation of the STORI for informal caregivers.

## Data Availability Statement

The data sets used and analyzed during the current study are available from the corresponding author on reasonable request.

## Ethics Statement

The studies involving human participants were reviewed and approved by the Human Research Ethics Committee of the Vaud Canton. The patients/participants provided their written informed consent to participate in this study.

## Author Contributions

CC-T, AC-P, and SR contributed to the conception and design of the study. CC-T contributed to data acquisition. CC-T and JF performed the statistical analysis and drafted the first manuscript. JF, AC-P, IG, and SR critically reviewed and revised the manuscript. All authors read and approved the final manuscript.

## Conflict of Interest

The authors declare that the research was conducted in the absence of any commercial or financial relationships that could be construed as a potential conflict of interest.

## Abbreviations

LOT–R, The Life Orientation Test–Revised; STORI, The Stages of Recovery Instrument; M, Mean; SD, Standard Deviation.
